# Iron gallic acid biomimetic nanoparticles for targeted magnetic resonance imaging

**DOI:** 10.1371/journal.pone.0306142

**Published:** 2024-07-02

**Authors:** Yan Chen, Zhaohui Zhang, Zhijian Chen, Shiqing Jiang, Aikebaier Reheman, Yifan Ouyang, Bo Yu, Qiuyan Chen, Dingtai Wei

**Affiliations:** 1 Fujian Key Laboratory of Toxicant and Drug Toxicology, Ningde Normal University, Ningde, Fujian, China; 2 Medical School, Ningde Normal University, Ningde, Fujian, China; 3 Department of Chemistry, Fuzhou University, Fuzhou, Fujian, China; 4 Functional and Molecular Imaging Laboratory for Cerebral Vascular Diseases, Ningde Municipal Hospital of Ningde Normal University, Ningde, Fujian, China; 5 Radiology Department, Ningde Municipal Hospital of Ningde Normal University, Ningde, Fujian, China; 6 Pharmacy Department, Ningde Municipal Hospital of Ningde Normal University, Ningde, Fujian, China; Hamadan University of Medical Sciences, ISLAMIC REPUBLIC OF IRAN

## Abstract

Developing T_1_-weighted magnetic resonance imaging (MRI) contrast agents with enhanced biocompatibility and targeting capabilities is crucial owing to concerns over current agents’ potential toxicity and suboptimal performance. Drawing inspiration from “biomimetic camouflage,” we isolated cell membranes (CMs) from human glioblastoma (T98G) cell lines via the extrusion method to facilitate homotypic glioma targeting. At an 8:1 mass ratio of ferric chloride hexahydrate to gallic acid (GA), the resulting iron (Fe)–GA nanoparticles (NPs) proved effective as a T_1_-weighted MRI contrast agent. T98G CM–coated Fe–GA NPs demonstrated improved homotypic glioma targeting, validated through Prussian blue staining and *in vitro* MRI. This biomimetic camouflage strategy holds promise for the development of targeted theranostic agents in a safe and effective manner.

## 1. Introduction

Magnetic resonance imaging (MRI) is a widely used noninvasive diagnostic tool [1]. Exogenous contrast agents are typically employed to enhance imaging sensitivity [2]. Among these, T_1_-weighted contrast agents are favored in clinical practice for their ability to produce brighter signals from surrounding tissues [3]. In comparison, T_2_-weighted contrast agents yield dark signals that are indistinguishable from naturally dark areas. Paramagnetic gadolinium(III)-chelated contrast agents, such as gadoteridol, are commonly used as T_1_ contrast agents, although concerns persist over their accumulation in the liver and kidneys [4, 5]. Therefore, the development of T_1_-weighted contrast agents with improved biocompatibility and targeting capabilities is urgently required.

MRI with targeting ability is pivotal for minimizing adverse effects and optimizing imaging performance [6, 7]. Therefore, cell membrane (CM)-coated nanoparticles (NPs), inspired by the concept of “biomimetic camouflage,” are garnering substantial attention [8, 9]. These extracellular vesicle–like NPs can not only evade immune detection but also target homotypic tumor sites [10, 11].

**Scheme 1:**
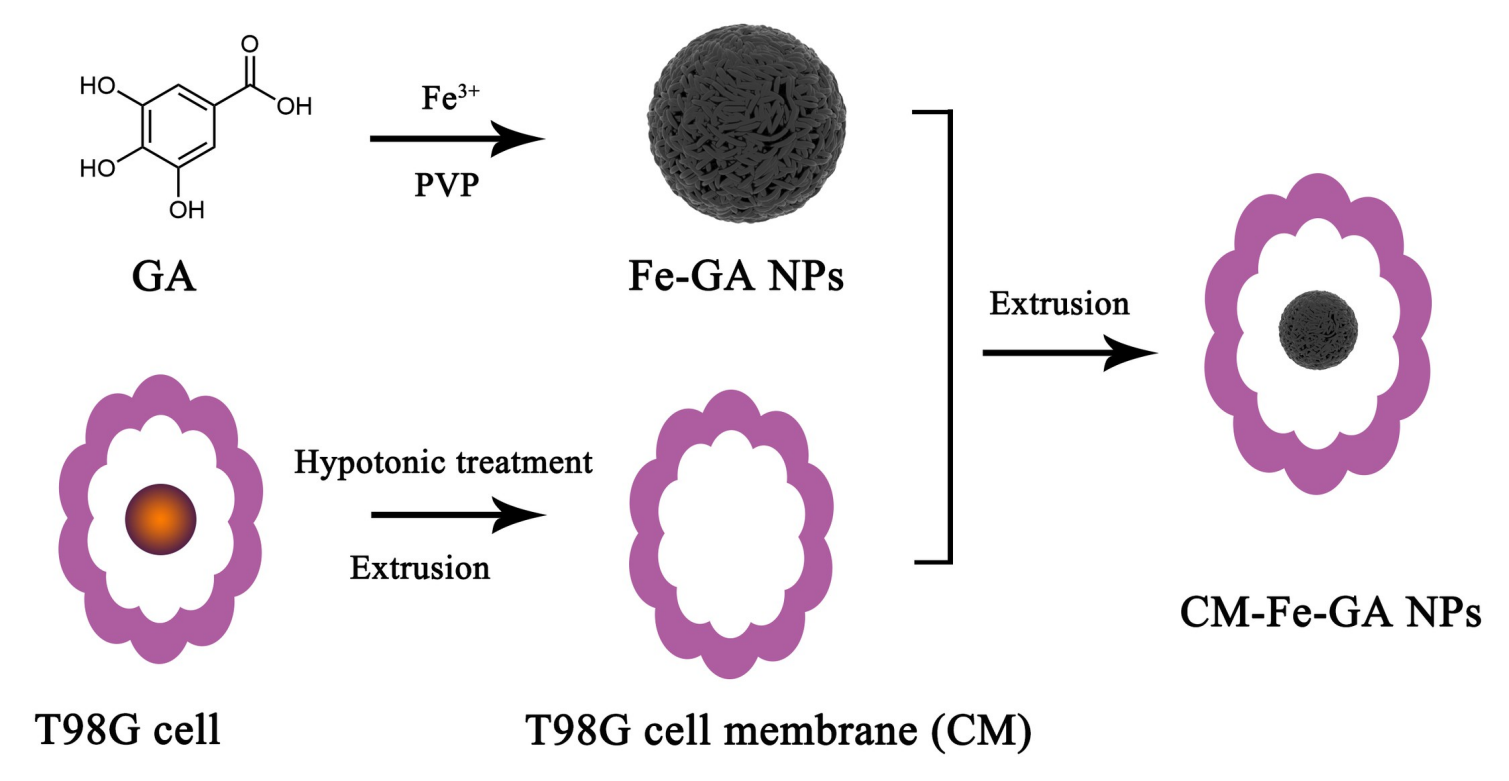
Schematic illustration for the synthesis of CM-Fe-GA NPs.

Consistent with this approach, we isolated CMs from human glioblastoma (T98G) cell lines, facilitating homotypic glioma targeting. Iron (Fe)-gallic acid (GA) NPs were introduced as MRI contrast agents, with T98G CM coating the NPs to enhance MRI targeting (Scheme 1). Fe, a component of the contrast agent, is less toxic than other paramagnetic ions, such as gadolinium and manganese [12]. Both polyvinylpyrrolidone (PVP) and GA, constituting the Fe–GA NPs, are US Food and Drug Administration–approved materials [13–15]. At an 8:1 mass ratio of ferric chloride hexahydrate (FeCl_3_·6H_2_O) to GA, Fe–GA NPs proved effective as T_1_-weighted MRI contrast agents. T98G CM–coated Fe–GA NPs (T98G CM-Fe–GA NPs), produced via the extrusion method, enabled T98G cell targeting, confirmed through Prussian blue staining and *in vitro* MRI. This biomimetic camouflage strategy will facilitate the development of targeted theranostic agents safely and effectively.

## 2. Materials and methods

### 2.1 Chemicals and characterization

Gallic acid (GA, analytical regent) and sucrose bought from Adamas. FeCl_3_·6H_2_O was purchased from Tianjing Hengxing chemical Co., Ltd. Polyvinylpyrrolidone (PVP, MW = 8000) was purchased from Macklin Biochemical Co., Ltd. Cell counting kit-8 (CCK-8) was obtained from Apexbio. Omni-easy instant bicinchoninic acid assay (BCA) protein assay kit was obtained from Yamei Co., Ltd. The Prussian Blue stain kit (eosin method) was obtained from Bioss. The deionized water was used in the experiments.

The atomic force microscope (AFM) micrographs were recorded on Bruker Dimension Icon (Germany). The DLS and zeta potential of the as-prepared samples were determined on a Zetasizer Nano ZS (Brookhaven Instruments Corporation). The elemental analysis was performed on an ICP-OES system (Agilent 720ES).

### 2.2 Synthesis of Fe-GA NPs

Colloidal Fe–phenolic coordination spheres were synthesized following a previously established procedure [12]. Briefly, PVP (0.1 g) was dissolved in 10 mL of water at room temperature until completely dissolved. Subsequently, 0.2 mL of FeCl_3_·6H_2_O (100 mg/mL) was added, followed by stirring for 1 h. GA (10 mg/mL) was then added to the solution, and stirring continued for 24 h. The resulting Fe–GA NPs were dialyzed against deionized water for 48 h (molecular weight cut-off: 14,000) and stored in a refrigerator for future use. Different mass ratios of FeCl_3_·6H_2_O to GA (2:1, 4:1, 8:1, 16:1, and 32:1) were achieved by adjusting the volume of GA added (1, 0.5, 0.25, 0.125, and 0.0625 mL, respectively) while maintaining the same initial Fe content (0.2 mL; 100 mg/mL FeCl_3_·6H_2_O).

### 2.3 Isolation of T98G cell membrane

T98G and mouse mononuclear macrophage (RAW 264.7) cell lines were cultured in Dulbecco’s modified Eagle medium (DMEM) supplemented with 10% fetal bovine serum and 1% penicillin–streptomycin (100×). T98G and RAW 264.7 CMs were obtained using the same protocol described previously [16]. Briefly, collected cells were suspended in TM buffer (pH 7.4; 10 mM Tris + 1 mM magnesium chloride) and extruded using a mini-extruder (without a polycarbonate membrane) at least 30 times. The cell suspension with 0.25 M sucrose was then centrifuged at 2000 *g* and 4°C for 10 min to remove cell debris. The CM was collected after the supernatant underwent continuous centrifugation at 3000 *g* for 30 min and was washed twice with ice-cold 0.25 M sucrose.

### 2.4 Preparation of CM-Fe-GA NPs

The Fe–GA NP solution was mixed with the CM suspension at a mass ratio of 1:0.28 (NPs to CMs), vigorously vortexed for 10 min, and then repeatedly extruded through pore sizes of 400 and 100 nm (polycarbonate membrane) using a mini-extruder (Avanti Polar Lipids), respectively. After 20 continuous extrusions, T98G CM-Fe–GA NPs were obtained.

### 2.5 Prussian blue stain for iron

The T98G cells were cultured in 6-well plates until reaching ∼80% confluence, washed with phosphate-buffered saline (PBS), and incubated with 2% bovine serum albumin for 30 min at 4°C. After washing with PBS for 3 times, the cells were incubated with either T98G CM-Fe–GA NPs or Fe–GA NPs (0.26 mg/mL in DMEM) for 4 h at 37°C. Following incubation, cells were washed three times with PBS, fixed with 4% paraformaldehyde for 5 min, and washed again with PBS. Ferric species were identified using a Prussian blue iron stain kit. A 1:1 mixture of Solution A:Solution B was added and incubated for 20 min. After rinsing with PBS, Solution C was added for 1 min. Following a PBS wash, cell coloration was observed using an inverted microscope (Olympus).

### 2.6 MRI of phantom

T98G cells were incubated in 100 mm Petri dishes and collected after incubation with T98G CM-Fe–GA NPs, RAW 264.7 CM-Fe–GA NPs, or Fe–GA NPs at a concentration of 0.24 mg/mL in PBS for 24 h at 37°C, respectively. The cells were detached with trypsin, washed with PBS, and centrifuged to collect cell pellets, which were immobilized in 1.5 mL tubes with 1% agarose. Unstained cells served as the control group.

T_1_-weighted magnetic resonance images were acquired using a SIGNA Architect 3.0T MRI scanner (United States) with the following parameters: repetition time (TR): 750 ms; echo time (TE): 7.6 ms; slice thickness: 3.0 mm. T_1_ relaxation times were quantified using MAGiC technology from GE Health Care (United States) with the following parameters: TR: 4000 ms; TE: 14 ms; Eff TE2: 84.3 ms; slice thickness: 3.0 mm.

### 2.7 Western blot

Western blotting was performed using a 1:200 dilution of primary polyclonal mouse anti-Aquaporin 4 (AQP4) antibody [AQP4 (4/18): sc-32739, Santa Cruz Biotechnology, Inc.] following the manufacturer’s instructions. Loading was standardized with a 1:1000 dilution of monoclonal mouse anti-GAPDH (5174S, Cell Signaling Technology).

### 2.8 Cytotoxicity assay

We seeded T98G cells in a 96-well plate and added Fe–GA NPs at various concentrations (0, 4, 8, 21.12, and 42.24 μg/mL). After a 24 h incubation at 37°C, culture media were removed, and each well received 100 μL of PBS and 10 μL of Cell Counting Kit-8 (CCK-8) agent. The absorbance at 450 nm was measured after 1 h of incubation at 37°C to evaluate the cytotoxicity of Fe–GA NPs.

## 3. Results

### 3.1. Synthesis and characterization

**Fig 1:**
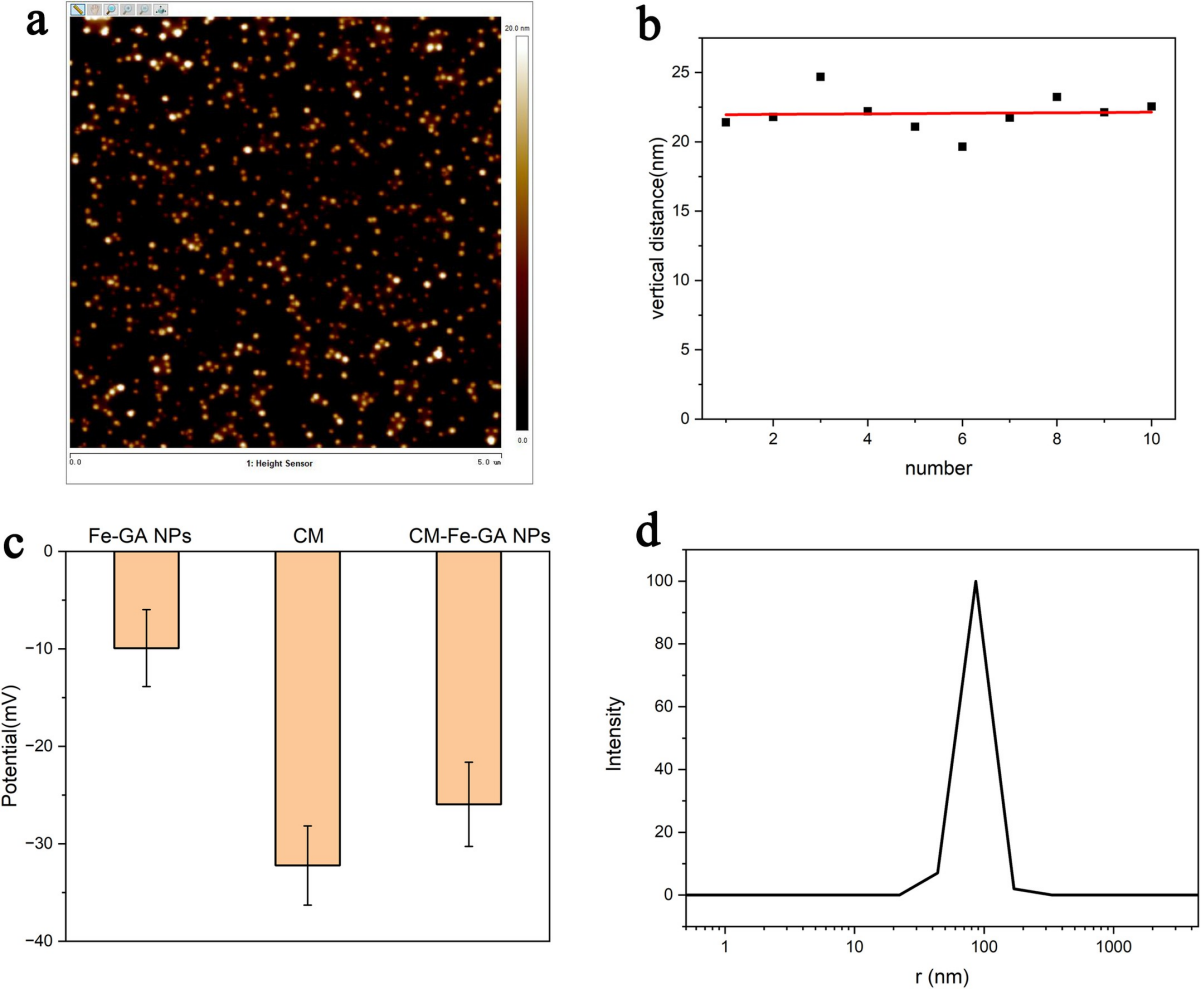
AFM images of Fe-GA NPs (a) and vertical distance of the nanoparticle measured from AFM images by software Nanoscope Analysis (b). The zeta potential of different groups (c), and DLS photograph of T98G CM-Fe-GA NPs (d).

The monodispersed coordination nanocomplex (Fe–GA NPs) was successfully prepared through the coordination between Fe and GA (S1 Fig). The diameter of Fe–GA NPs was determined by the measuring the vertical distance from atomic force microscopy images, yielding a mean value of 22.05 nm (Fig 1A and 1B).

T98G cells were extracted through a hypotonic treatment and centrifugated to isolate CMs. Total protein content was assessed using a bicinchoninic acid protein assay. The purified CMs were mixed with Fe–GA NPs and extruded through the polycarbonate membranes to produce T98G CM-Fe–GA NPs. As shown in Fig 1C, the mean zeta potential of Fe–GA NPs was measured at -9.92 mV, and the purified protein (T98G CM) exhibited a negative charge (mean value: -32.22 mV). After CM decoration, the mean hydrodynamic diameter was 72.22 nm, with a mean zeta potential of -25.95 mV (Fig 1C and 1D). Furthermore, Fe–GA NPs exhibited negligible cytotoxicity, as assessed via CCK viability testing (S2 Fig).

### 3.2. MRI performance

**Fig 2:**
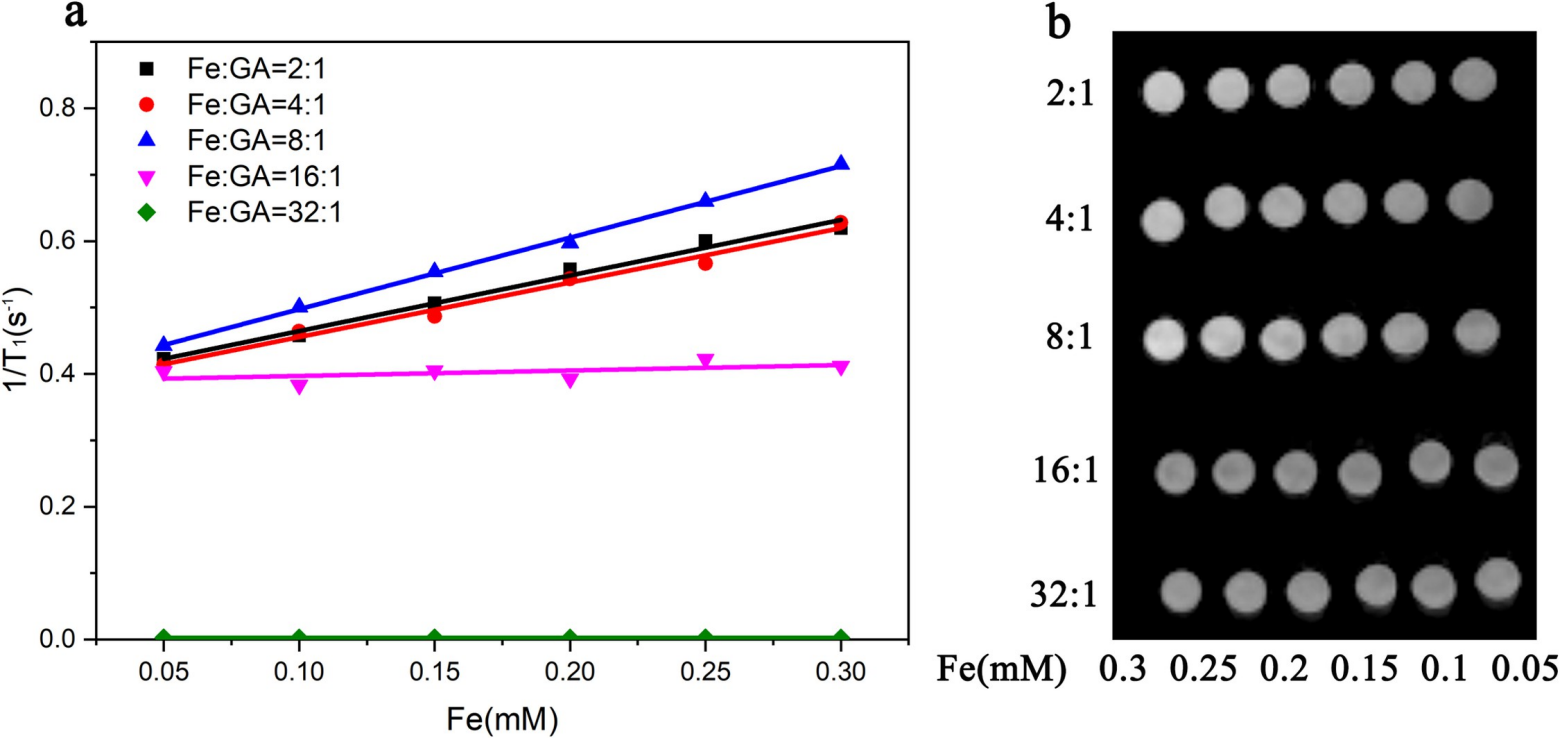
The relaxation rates (R_1_) versus different concentrations of iron (a) and T_1_ MR images (b) of Fe-GA NPs at different mass ratio of FeCl_3_·6H_2_O to GA.

To optimize T_1_-weighted relaxivity (r_1_), we explored various mass ratios of FeCl_3_·6H_2_O to GA: 2:1, 4:1, 8:1, 16:1, and 32:1. As shown in Fig 2, reciprocal values of relaxation time were plotted as a function of Fe concentration, with the slope of the fitted line representing the r_1_ value (Table 1). The precursor FeCl_3_·6H_2_O to GA mass ratio of 8:1 yielded the highest r_1_ value (r_1_ = 1.0777 mM^−1^ s^−1^); therefore, this mass ratio was used for further study. Visual examination of Fe–GA NP aqueous solutions revealed bleaching in samples with precursor mass ratios of 16:1 and 32:1 compared with other ratios (S3 Fig).

**Table 1:** The r_1_ value at different mass ratio of FeCl_3_·6H_2_O to GA.

The mass ratio of FeCl_3_·6H_2_O to GA	r_1_(mM^-1^ s^-1^)	Adj. R-Square
2:1	0.8369	0.9841
4:1	0.8202	0.9824
8:1	1.0777	0.9978
16:1	0.0820	0.1473
32:1	0.0001	-0.2400

### 3.3. Targeted MRI

**Fig 3:**
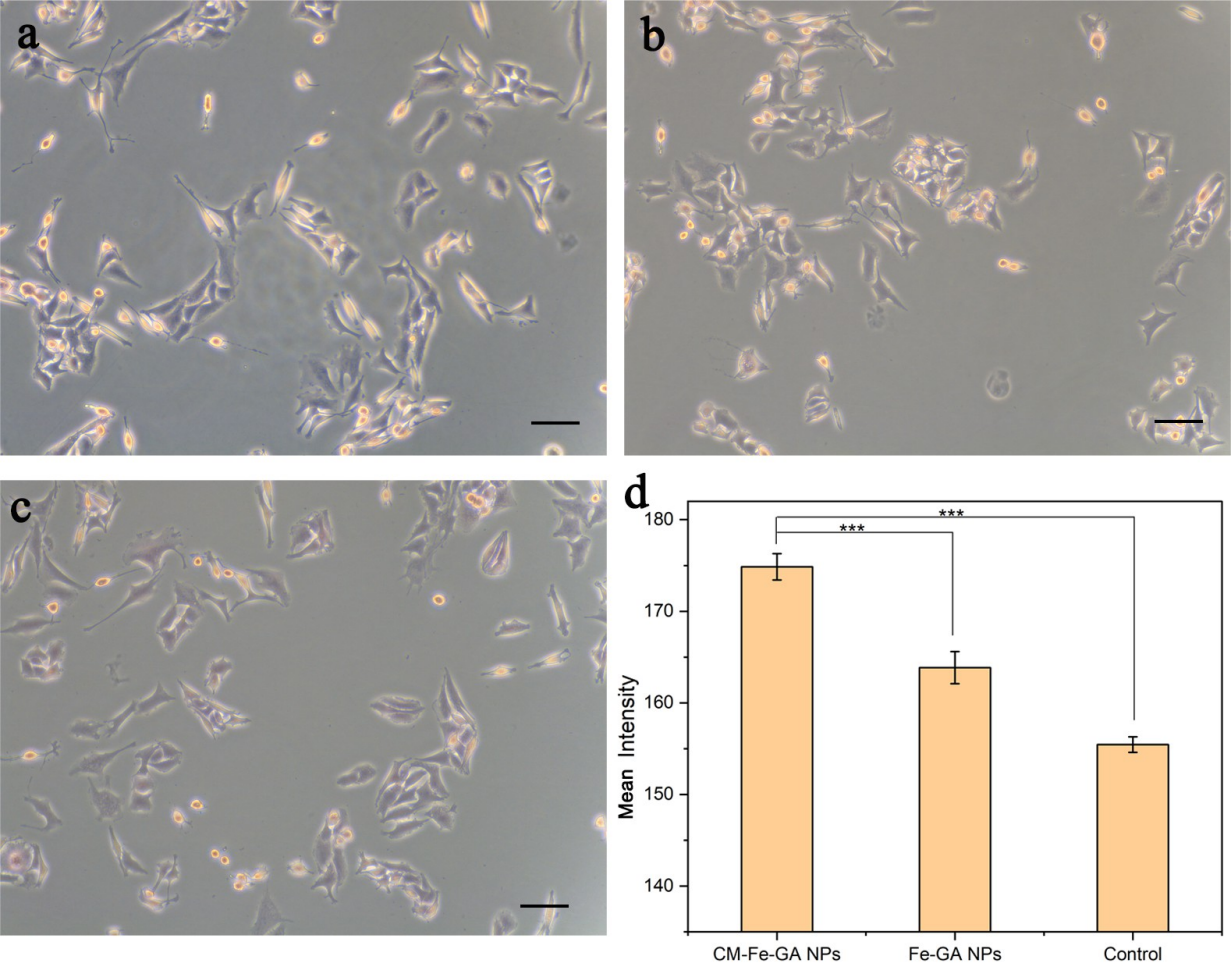
Specific binding of targeted nanoprobe in vitro for T98G cell. On Prussian blue stain, (a) T98G CM-Fe-GA NPs shows strong binding to cell surface of T98G cells by comparison with (b) Fe-GA NPs and (c) control. (d) Quantified mean intensities of the blue color by the software Image J. The error bars indicate the s.d. (n = 5, ***P<0.001 from an analysis of variance with two-tailed t test).

Initially, Prussian blue staining was performed on the T98G cell line, and blue Fe staining was quantified using Image J software (Fig 3). The T98G CM-Fe–GA NPs group showed greater Fe accumulation compared with the Fe–GA NPs or control groups, indicating the crucial role of T98G CMs in promoting cellular uptake of NPs.

**Fig 4:**
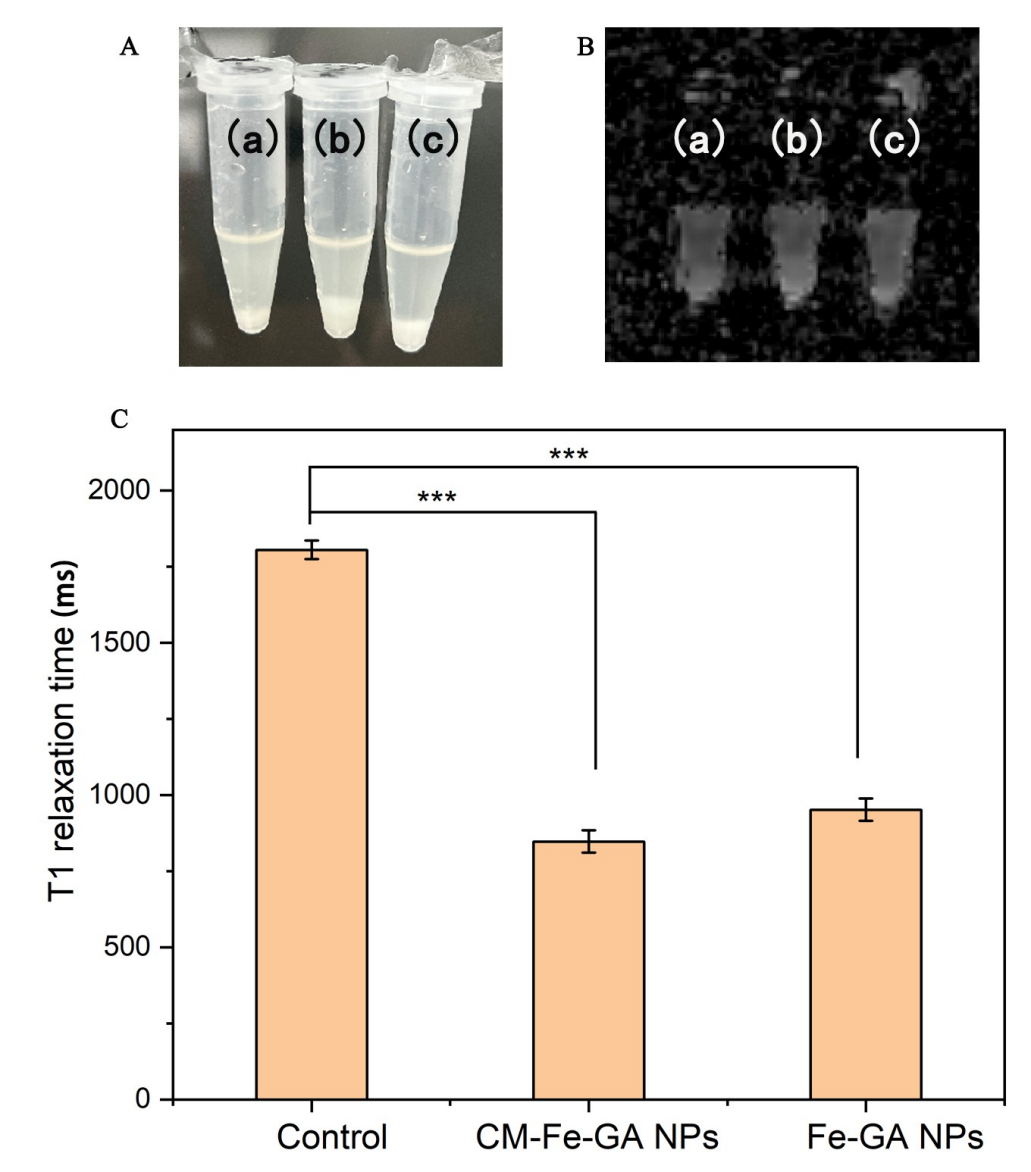
The photographs (A), T_1_-weighted MR images (B), and T_1_ relaxation time (C) of T98G cells incubated with T98G CM-Fe-GA NPs (a), Fe-GA NPs (b) and control group (c). The error bars indicate the s.d. (n = 3, ***P<0.001 from an analysis of variance with two-tailed t test).

Subsequently, we assessed the targeting ability of T98G CM-Fe–GA NPs against T98G cell lines using T_1_-weighted MRI. The mean T_1_ relaxation time for T98G CM-Fe–GA NPs was shortened compared to cells incubated with either Fe–GA NPs or the control (Fig 4). Additionally, T_1_ relaxation times were evaluated by comparing T98G CM-Fe–GA NPs and RAW264.7 CM-Fe–GA NPs against T98G cells. Results revealed that T98G CM-Fe–GA NPs exhibited lower T_1_ relaxation times compared with RAW264.7 CM-Fe–GA NPs or the control group (S4 and S5 Figs). The T98G CM coating facilitated the entry of the T_1_-weighted contrast agent (T98G CM-Fe–GA NPs) into T98G cells owing to its homotypic targeting ability, resulting in a marked reduction in T_1_ relaxation time.

## 4. Discussion

Following a previously established method, Fe–GA NPs were successfully synthesized through a one-step coordination process. GA, a typical type of tea polyphenol, possesses phenolic hydroxyl and carboxyl groups that can coordinate with Fe^3+^ to form the coordination nanocomplex at room temperature. PVP serves as both a protective polymer for the coordination nanocomplex and a chelating agent to Fe^3+^. The absolute zeta potential value of T98G CM-Fe–GA NPs fell between that of Fe–GA NPs and T98G CMs (Fig 1C), indicating successful coating with T98G CMs. Furthermore, with ultralow cell toxicity and paramagnetic properties, Fe–GA NPs have the potential to serve as a safe T_1_-weighted MRI contrast agent.

T_1_-weighted magnetic resonance images of Fe–GA NPs were obtained in aqueous solutions with varying Fe content, revealing the brightest T_1_-MRI signal at a precursor mass ratio of 8:1. It appeared that the Fe content of Fe–GA NPs markedly influenced T_1_-weighted MRI performance. Excess Fe precursors failed to coordinate with GA to form Fe–GA NPs. The coordinated Fe–GA NPs enhanced the mobility of coordinated water around the Fe paramagnetic center, thereby improving r_1_ values [17].

The presence of T98G CMs on the surface of CM-Fe–GA NPs facilitates NP escape from the reticuloendothelial system, targets gliomas cells, promotes cellular uptake, and holds great potential for targeted MRI of gliomas. Gliomas are primary brain tumors thought to derive from neuroglial stem or progenitor cells [18]. AQP4, a major water channel protein, plays a key role in glioma development [19], and abnormal upregulation of AQP4 expression may serve as an indicator of glioma, facilitating glioma infiltration into the brain [20]. Thus, AQP4 is a potential target for early glioma detection using targeted MRI. Although the transmembrane water-efflux rate measured via MRI can serve as a biomarker for AQP4 in gliomas [21], exogenous contrast agents targeting AQP4 have not been reported. Western blot results revealed high AQP4 expression in the T98G cell line (S6 Fig). Therefore, the developed T98G CM-Fe–GA NPs show promise as an AQP4-targeted MRI contrast agent.

Although our results are promising, further investigations of the MRI-targeted nanoprobe in animals are warranted before clinical translation. Our next step will involve expanded studies in a larger number of animals to ensure safety and effectiveness prior to human use. These modifications will help validate our targeted nanoprobe for early detection of gliomas using MRI.

## 5. Conclusion

In this study, we report the successful synthesis and characterization of T98G CM-Fe–GA NPs as a targeted MRI contrast agent for gliomas. The NPs were synthesized using a one-step coordination process, incorporating GA and PVP. We found that T98G CM-Fe–GA NPs exhibit enhanced cellular uptake and accumulation in glioma cells compared with Fe–GA NPs, as confirmed by Prussian blue staining and T_1_-weighted MRI. Our results highlight the potential of T98G CM-Fe–GA NPs as a promising tool for targeted MRI of gliomas. These primary brain tumors have limited treatment options and poor prognosis, posing major challenges in early detection and targeted therapy. The ability of T98G CM-Fe–GA NPs to specifically target glioma cells while exhibiting minimal cytotoxicity and excellent T_1_-weighted MRI contrast enhancement offers new opportunities for early diagnosis and personalized treatment strategies. Overall, we believe that our findings make a significant contribution to the field of targeted molecular imaging and hold promise for improving the diagnosis and management of gliomas.

## Supporting information

S1 Fig TEM images of Fe-GA NPs (a) and CM-Fe-GA NPs (b-c).
(DOCX)

S2 Fig Viability of T98G cell in the presence of Fe-GA NPs. (DOCX)

S3 Fig Photographs of Fe-GA NPs at different mass ratio of FeCl_3_·6H_2_O to GA (Concentration of iron: 0.3mM). (DOCX)

S4 Fig The T_1_ relaxation time of T98G cell incubated with T98G CM-Fe-GA NPs, RAW264.7 CM-Fe-GA NPs and control groups. The error bars indicate the s.d. (n = 3, *P<0.05 from an analysis of variance with two-tailed t test).
(DOCX)

S5 Fig The T_1_-weighted MR images of T98G cells incubated with control group (a), RAW264.7 CM-Fe-GA NPs (b), and T98G CM-Fe-GA NPs (c). The red square indicated cell pellets at the bottom of the tube.
(DOCX)

S6 Fig Western Blot of T98G cell for AQP4 at the cell protein content of 16.4ug (a) and 53.8ug (b).
(DOCX)
