# Supplementary material for: Iron gallic acid biomimetic nanoparticles for targeted magnetic resonance imaging
Source: PLoS One. 2024 Jul 2;19(7):e0306142. doi: 10.1371/journal.pone.0306142 (PMC11218937; doi:10.1371/journal.pone.0306142)
Supplement: S2 Fig — (DOCX) [file pone.0306142.s002.docx]

**Iron gallic acid biomimetic nanoparticles for targeted magnetic resonance imaging**





Fig. S2. Viability of T98G cell in the presence of Fe-GA NPs.
