# Supplementary material for: Iron gallic acid biomimetic nanoparticles for targeted magnetic resonance imaging
Source: PLoS One. 2024 Jul 2;19(7):e0306142. doi: 10.1371/journal.pone.0306142 (PMC11218937; doi:10.1371/journal.pone.0306142)
Supplement: S4 Fig — The error bars indicate the s.d. (n = 3, *P<0.05 from an analysis of variance with two-tailed t test). (DOCX) [file pone.0306142.s004.docx]

**Iron gallic acid biomimetic nanoparticles for targeted magnetic resonance imaging**





Fig. S4. The T_1_ relaxation time of T98G cell incubated with T98G CM-Fe-GA NPs, RAW264.7 CM-Fe-GA NPs and control groups. The error bars indicate the s.d. (n=3, *P<0.05 from an analysis of variance with two-tailed t test).
