# Supplementary material for: Iron gallic acid biomimetic nanoparticles for targeted magnetic resonance imaging
Source: PLoS One. 2024 Jul 2;19(7):e0306142. doi: 10.1371/journal.pone.0306142 (PMC11218937; doi:10.1371/journal.pone.0306142)
Supplement: S5 Fig — The T1-weighted MR images of T98G cells incubated with control group (a), RAW264.7 CM-Fe-GA NPs (b), and T98G CM-Fe-GA NPs (c). The red square indicated cell pellets at the bottom of the tube. (DOCX) [file pone.0306142.s005.docx]

**Iron gallic acid biomimetic nanoparticles for targeted magnetic resonance imaging**


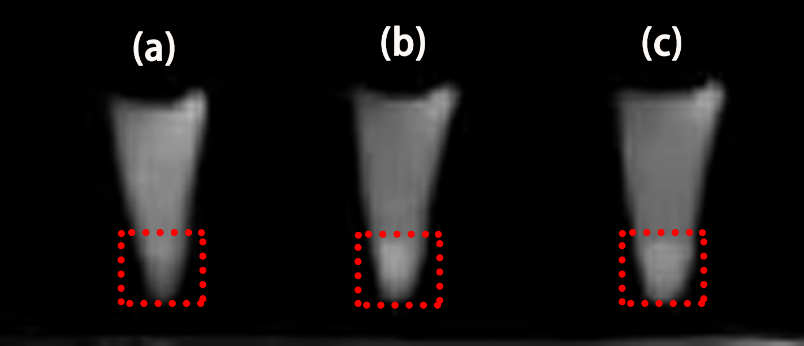


Fig. S5. The T_1_-weighted MR images of T98G cells incubated with control group (a), RAW264.7 CM-Fe-GA NPs (b), and T98G CM-Fe-GA NPs (c). The red square indicated cell pellets at the bottom of the tube.
