# Supplementary material for: Iron gallic acid biomimetic nanoparticles for targeted magnetic resonance imaging
Source: PLoS One. 2024 Jul 2;19(7):e0306142. doi: 10.1371/journal.pone.0306142 (PMC11218937; doi:10.1371/journal.pone.0306142)
Supplement: S6 Fig — Western Blot of T98G cell for AQP4 at the cell protein content of 16.4ug (a) and 53.8ug (b). (DOCX) [file pone.0306142.s006.docx]

**Iron gallic acid biomimetic nanoparticles for targeted magnetic resonance imaging**


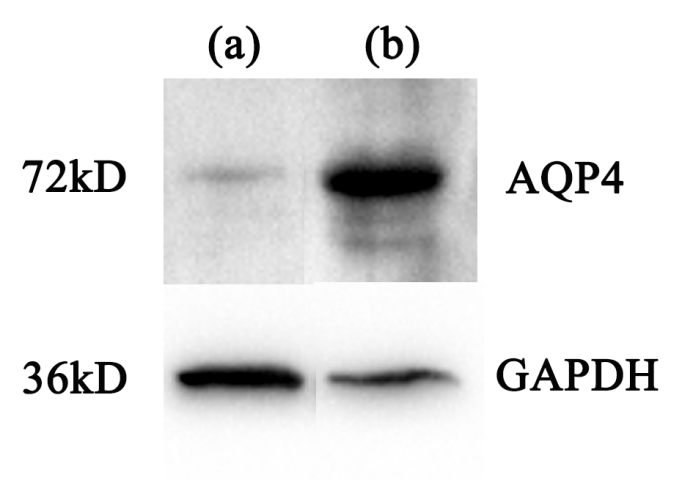


Fig. S6. Western Blot of T98G cell for AQP4 with the cell protein content of 16.4ug (a) and 53.8ug (b).
